# Retraction Note to: LISPRO mitigates β-amyloid and associated pathologies in Alzheimer’s mice

**DOI:** 10.1038/s41419-021-04444-7

**Published:** 2021-12-09

**Authors:** Ahsan Habib, Darrell Sawmiller, Song Li, Yang Xiang, David Rongo, Jun Tian, Huayan Hou, Jin Zeng, Adam Smith, Shengnuo Fan, Brian Giunta, Takashi Mori, Glenn Currier, Douglas Ronald Shytle, Jun Tan

**Affiliations:** 1 Rashid Laboratory for Developmental Neurobiology, Silver Child Development Center, Department of Psychiatry and Behavioral Neurosciences, Morsani College of Medicine, University of South Florida, Tampa, FL USA; 2 Center of Excellence for Aging and Brain Repair, Department of Neurosurgery and Brain Repair, Morsani College of Medicine, University of South Florida, Tampa, FL USA; 3 Departments of Biomedical Sciences and Pathology, Saitama Medical Center and Saitama Medical University, Kawagoe, Saitama Japan

Retraction Note to: *Cell Death & Disease* 10.1038/cddis.2017.279

The Editors have retracted this article because an investigation by the University of South Florida concluded that Figs. 7b, e and 8c–e present inappropriately duplicated images and that these images and the text associated with them are not scientifically defensible. Adam Smith, Brian Giunta, Glenn Currier, and Douglas Ronald Shytle agree with this retraction. Ahsan Habib, Darrell Sawmiller, and Takashi Mori disagree with this retraction. Jun Tan, Yang Xiang, David Rongo, Huayan Hou, and Song Li have not responded to any correspondence from the publisher about this retraction. The publisher was unable to contact Jun Tian, Jin Zeng, and Shengnuo Fan.

